# The nightmare of catheter ablation in a young male with incessant supraventricular tachycardia

**DOI:** 10.1002/joa3.13120

**Published:** 2024-07-23

**Authors:** Dat Cao Tran, Chin‐Yu Lin

**Affiliations:** ^1^ Taipei Veteran General Hospital Taipei Taiwan; ^2^ Cho Ray Hospital Ho Chi Minh city Vietnam; ^3^ Department of Medicine National Yang‐Ming Chiao‐Tung University Taipei Taiwan

**Keywords:** ablation, accessory pathway, coronary spasm, electrophysiology study

## Abstract

A case report involving incessant multi‐types of supraventricular tachycardia and acute decompensated heart failure required a rescuing electrophysiology study and ablation. Ventricular fibrillation occurred due to coronary spasm, complicating the deteriorating heart. However, aggressive therapies, including extracorporeal support and the timely elimination of the culprit accessory pathway, successfully resolved the patient's condition.

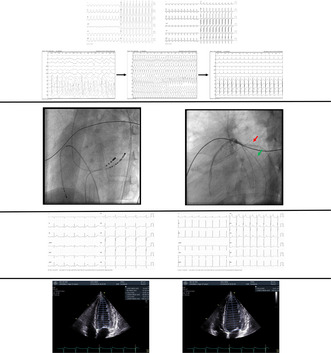

A 17‐year‐old male patient was transferred to our hospital due to a sudden collapse in the other hospital. The patient was found to suffer from frequent palpitation and light‐headedness that eventually led to his collapse. At the previous hospital, different supraventricular tachycardias (Figure 1) were documented. As the patient initial presented with tachycardia and narrow QRS, aggressive medical treatment with multiple repeated doses of adenosine (12 mg) was given to terminate the arrhythmia. However, the supraventricular tachycardia (SVT) spontaneously re‐initiated despite additional maintenance therapy with intravenous amiodarone and his condition deteriorated significantly with eventual seizure and cardiac arrest with pulseless electrical activity (PEA) (Figure 2). SVT still persisted follow successful resuscitation, and after half a day, the patient was transferred to our hospital. The bedside reexamination echocardiography revealed a left ventricular ejection fraction (LVEF) of 10%. An emergency coronary angiography and electrophysiology study were then performed which confirmed the intact coronary arteries and diagnosis of orthodromic atrioventricular tachycardia (AVRT) with a left lateral accessory pathway (AP) and the concomitant atrial tachycardia. As radiofrequency energy was being delivered to eliminate the accessory pathway via the retrograde approach (Figure 3), the progressive changes of the ST interval over the precordial leads were observed. Within a few seconds, the sinus rhythm deteriorated into ventricular fibrillation (Figure 4 Panel A). Defibrillation with cardio‐pulmonary resuscitation was immediately performed. Under the suspicion of coronary injury or spasm, a repeat angiogram was performed, which showed an intact coronary system. As the patient's condition deteriorated dramatically, we put him on extracorporeal support and stopped the procedure with partial success in controlling the tachycardia.

**FIGURE 1:**
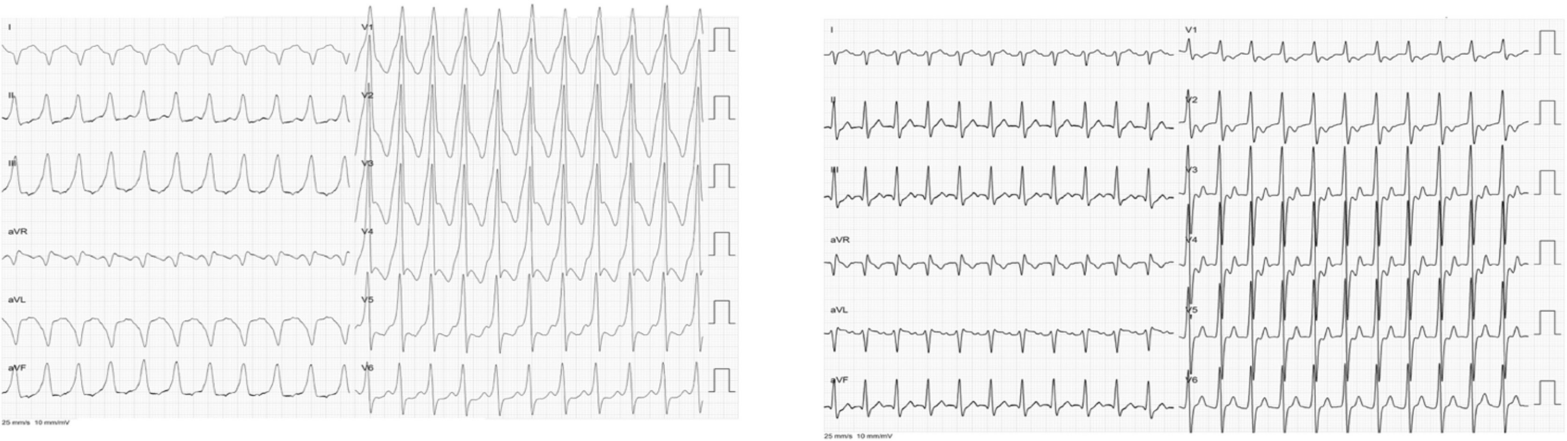
Different supraventricular tachycardia presentations of the patient at admission. Left panel: Atrial tachycardia with preexcitation (wide QRS with the delta wave). Right panel: Orthodromic atrioventricular reentry tachycardia.

**FIGURE 2:**
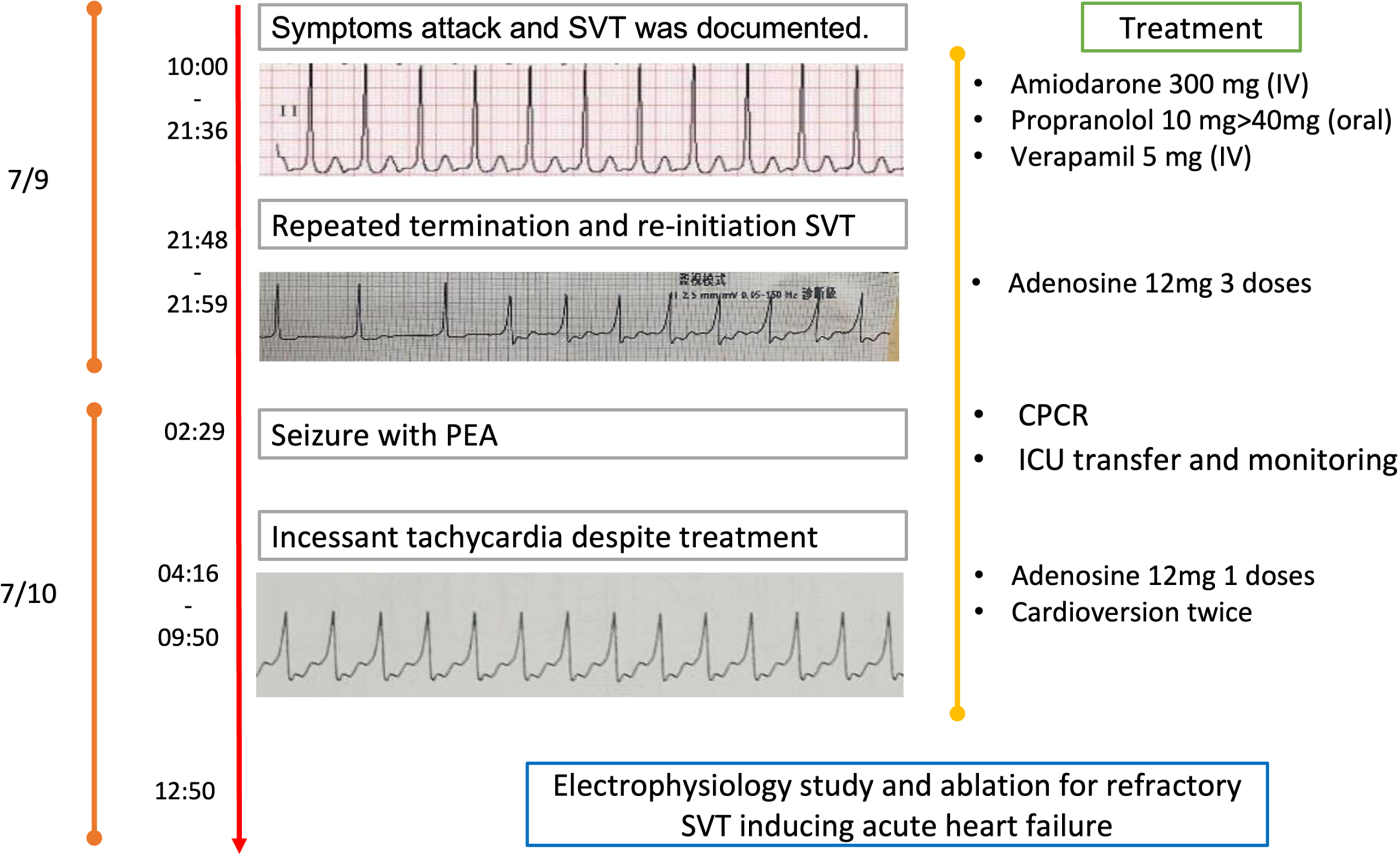
Timeline for the patient's progress and treatment. The progression of the tachycardia from one with narrow QRS to one with wide QRS (possibly tachycardia with preexciation).

**FIGURE 3:**
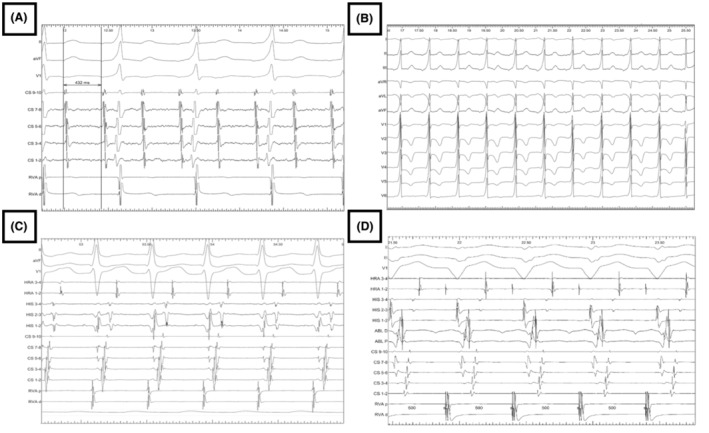
Intracardiac echocardiogram during the electrophysiology study and ablation. Panel A Intracardiac echocardiogram of the atrial tachycardia (AT) with proximal to distal atrial activation and atrioventricular (AV) 2 to 1 conduction with preexcitation. Panel B 12 leads ECG of the AT. Panel C AVRT with the eccentric distal to proximal CS atrial activation ventricular pacing with eccentric atrial activation. Panel D intracardiac signal at the successful ablation site for the AP during the right ventricular pacing.

**FIGURE 4:**
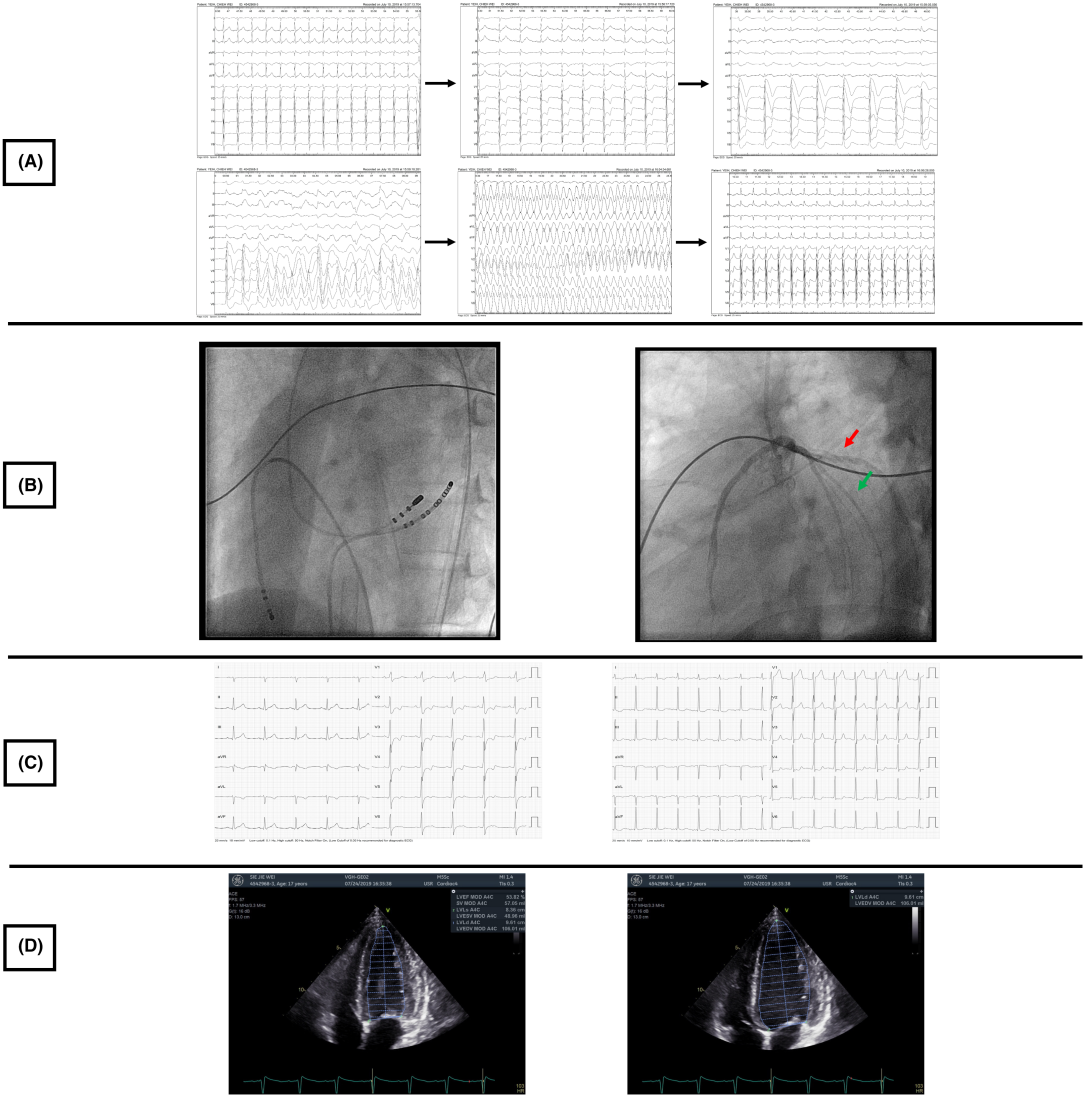
ECG during the ablation procedure. Panel A ST‐T changed with depression of the V1‐V3 then elevation and sinus rhythm progressed to VF. Panel B Fluoroscopic view of the ablation site and coronary angiogram of the left coronary in correlation with the ablation site (Red arrow: Left circumflex coronary, Green arrow: Diagonal branch). Panel C: Sinus rhythm with preexcitation before ablation and the normal sinus rhythm without preexcitation after ablation. Panel D: Echocardiography after the ablation with normalization of the EF (52%).

The patient presented to the hospital with incessant refractory tachycardia, acute decompensated heart failure symptoms, and sudden cardiac arrest. Since the patient presented with PSVT and the narrow QRS, the initial treatment with adenosine was well justified. However, since the SVT was then shifted to the one with wider QRS, the usage of antiarrhythmic medication with longer action duration should be exercised carefully as amiodarone might reduce the nodal conduction and this effect would be fatal in the case of SVT using the accessory pathway as the atrioventricular conduction. It is not uncommon for arrhythmias to cause acute heart failure, and the diagnosis was further supported by the elevated NT‐proBNP (364 pg/mL) and the reduced ejection fraction of only 10% at our emergency department. Tachycardia‐induced heart failure has emerged in recent years as one common cause of acute decompensated heart failure, and patients also run a higher risk of sudden cardiac death.^1^ The incessant tachycardia in our case might be the culprit for the acute heart failure as evidenced by the complete recovery of the LV function after the tachycardia was eliminated. The tachycardia that had led to heart failure was most likely both orthodromic and antidromic forms of AVRT, and later on, another diagnosis was found during our EP study was the AT with a by‐stander accessory pathway. In such scenario, the AP has an important role as it would contribute to the former arrhythmia and facilitate the fast ventricular rate in the latter tachycardia. As we considered the AP with short refractory period would underlie the formation of tachycardia and the acute heart failure, we aimed to achieve complete elimination of the pathway and no AT was induced afterward. After the episode of ventricular fibrillation, amiodarone 200 mg twice per day was prescribed after loading dose for 24 h. The AT might be suppressed by amiodarone. With all of the prior incessant forms of even supraventricular tachycardia, the left ventricular function would deteriorate significantly in such a short period of time. However, the procedure was further complicated by ventricular fibrillation (VF). ST elevation before the lethal arrhythmia, the location of the left lateral portion of the mitral annulus, and the normal coronary angiogram that followed yielded the suspicion of left circumflex coronary spasm. Coronary spasm is one rare yet still‐existing complication of catheter ablation, which could sometimes lead to catastrophic events.^2^, ^3^ As for the cause of coronary injury, direct thermal assault would be most likely and has been reported to cause adjacent vessels spasm. This complication has been reported in some previous studies in the pediatric patients with relatively thin myocardium as the predisposing factor.^4^ For the group of older patients, the incidence seems to be significantly lower and ablation of the left AP is relatively safe and never before had there been a report discussing the acute dilation of the heart in adult patients as the predisposing factor for coronary injury during ablation. In the case, the patient's risk factor would be different but also at the same time seem to be similar with the acutely dilated ventricle resulting in the thinning of the myocardium wall, energy might have more close proximity with the coronary. With the use of only 4‐mm catheter, heat would accumulate higher and spread over the thinned myocardium which might affect the adjacent coronaries. It is advisory to point out that the patient had an acutely dilated ventricle which renders its completely different from the normal anatomy. In addition, the reduced coronary flow during fast heart rate and the acutely failing systolic function renders the heart more propensity for ventricular arrhythmias such as VT/VF. In the following days, with the tachycardia successful control with the emergency ablation, the patient improved gradually with EF increase from the initial 10%–30% and eventually normalized at 52% with no residual hypokinesis. However, intermittent preexcitation was still documented on his continuous ECG monitoring, but no episode of tachycardia was recorded. A cardiac MRI was then taken to rule out acute myocarditis, which showed a mildly dilated left ventricle with no sign of acute inflammation. A second electrophysiology study was then performed, and we completely ablated the accessory pathway without any complication. As we considered the AP with short refractory period would underlie the formation of tachycardia and the acute heart failure, we aimed to achieve complete elimination of the pathway and no AT was induced afterward. The patient was discharged and scheduled for a revisit at our outpatient clinic. After 1 year, he presented with an episode of palpitation with documented narrow QRS tachycardia. The third procedure was consulted for him, during which the supraventricular tachycardia was revealed to be focal atrial tachycardia which suspected to be the same AT in the first procedure. Due to unavailability of the discernable P wave morphology during the first procedure, we cannot make the complete correlation. Furthermore, in the initial admission, the response of the tachycardia to adenosine was termination and reinitiating so AT with reentry mechanism was the least likely. After the episode of ventricular fibrillation, amiodarone 200 mg twice per day was prescribed after loading dose for 24 h. The AT might be suppressed by amiodarone. During the third procedure, focal ablation over the origin of the SVT at the high crista terminalis terminates the tachycardia and renders it uninducible.

Incessant tachycardia could lead to acute decompensated heart failure and emergency electrophysiology study ablation could help delineate the cause and remained the rescue therapy in these scenarios where conservative medical treatment had failed to control the arrhythmias. Performing procedures in this group of patients would carry increasing risks due to the acutely dilated and thinned myocardium as well as the reduced blood flow to the coronary. Coronary spasm is one rare yet still‐existing complication of catheter ablation, which could sometimes lead to catastrophic events. However, with rigorous treatment therapies, these scenarios could be reversible and successfully managed.

## CONFLICT OF INTEREST STATEMENT

Authors declare no conflict of interests for this article.

## Supporting information


Figure S1.
