# Supplementary figures and images for: The nightmare of catheter ablation in a young male with incessant supraventricular tachycardia
Source: J Arrhythm. 2024 Jul 23;40(5):1183–6. doi: 10.1002/joa3.13120 (PMC11474928; doi:10.1002/joa3.13120)

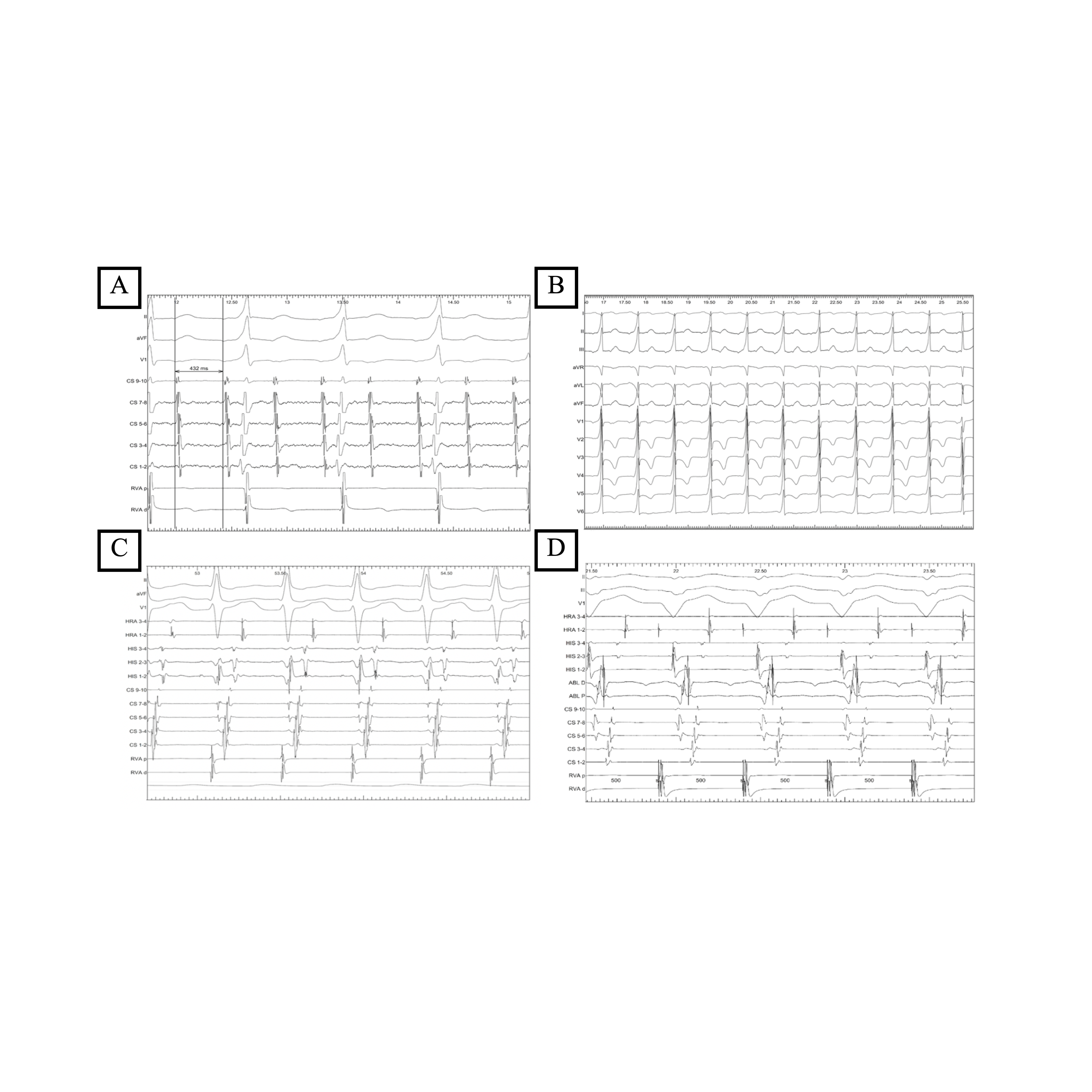

Supplement: Supplementary file 1 — Figure S1. [file JOA3-40-1183-s001.png]
